# The Cytotoxic Activity of Natural Killer Cells Is Suppressed by IL-10^+^ Regulatory T Cells During Acute Retroviral Infection

**DOI:** 10.3389/fimmu.2018.01947

**Published:** 2018-08-27

**Authors:** Elisabeth Littwitz-Salomon, Anna Malyshkina, Simone Schimmer, Ulf Dittmer

**Affiliations:** Institute for Virology, University Hospital Essen, University of Duisburg-Essen, Essen, Germany

**Keywords:** natural killer cells, regulatory T cells, friend retrovirus, IL-10, immune suppression, TGF-β

## Abstract

Natural killer (NK) cells play a key role in host defense against cancer and viral infections. It was shown that NK cells are important for the control of acute retroviral infections, but their antiviral activity depends on multiple parameters such as viral inoculation dose, interactions with myeloid cell types and the cytokine milieu. In addition, during an ongoing retroviral infection regulatory T cells (Tregs) can suppress NK cell functions. However, the precise role of Tregs on the initial NK cell response and their immediate antiviral activity after an acute retroviral infection is still unknown. Here we show that thymus-derived Tregs suppress the proliferation, effector functions and cytotoxicity of NK cells very early during acute Friend Retrovirus (FV) infection. Tregs exhibited an activated phenotype and increased the production of the immunosuppressive cytokines IL-10 and TGF-β after FV infection of mice. Neutralization of the immunosuppressive cytokine IL-10 resulted in a significant augmentation of NK cell functions. Although the activation of dendritic cells (DCs) and macrophages as well as the IL-15 cytokine levels were increased after Treg depletion, Tregs mainly affect the NK cell activity in an IL-10-regulated pathway. In this study we demonstrate an IL-10-dependent suppression of NK cells by activated Tregs during the first days of a retroviral infection.

## Introduction

Natural killer (NK) cells are very important for the control of viral infections. NK cells belong to the innate immune system and express germline-encoded receptors. They eliminate cancer cells and virus-infected cells via different pathways and without prior sensitization. Upon target cell identification and activation, NK cells release cytotoxic granules, containing granzymes and perforin, into immunological synapsis to induce the apoptosis of target cells (1). NK cells also express and secrete death receptor ligands such as Tumor Necrosis Factor Related Apoptosis Inducing Ligand (TRAIL) and FasL, which can also result in cell death of infected cells. Furthermore, a repertoire of cytokines such as interferon (IFN) γ, tumor necrosis factor α (TNFα), granulocyte-macrophage colony-stimulating factor (GM-CSF) can be produced and released by NK cells, which can mediate direct antiviral functions or influence adaptive immune responses (2). The antibody-dependent cell-mediated cytotoxicity (ADCC) is another mechanism of NK cells to eliminate infected cells. Here FcγRIII of NK cells recognize the constant region of antibodies and eliminate antibody-opsonized cells. NK cell activation and expansion during viral infections strongly depend on the cytokine milieu and the interaction with other immune cells (3, 4). Especially IFNα, Interleukin (IL)-2, IL-12, IL-15 and IL-18 play important roles for the survival, proliferation and activation of NK cells (2, 5). IL-2 is mainly produced by activated CD4^+^ T cells (6, 7), whereas macrophages and dendritic cells (DCs) can produce several NK cell-stimulatory cytokines (8, 9) and contact-dependent interactions between these myeloid cells and NK cells have been shown to activate NK cells (10–12).

The immediate activation of NK cells is very important for the efficient control of viral infections (13, 14). Viruses such as influenza viruses, herpes viruses or retroviruses cause a number of health issues. For some of these viral pathogens vaccinations or treatment is feasible, but for others it is still not possible to prevent or cure the infection. Major health problems are caused by the human immunodeficiency virus (HIV). The anti-retroviral therapy (ART), currently used for the treatment of HIV patients, suppresses viral replication; however, a cure is still impossible. Therefore, more basic research is necessary to find a way to cure or functionally cure HIV infections and prevent the onset of Acquired Immunodeficiency Syndrome (AIDS)-associated disease. The Friend retrovirus (FV) mouse model is a well-established model for the analysis of immune cell responses during retroviral infection, although it does not resemble the pathological features of HIV-1 infection. FV replicates in the bone marrow, spleen and lymph nodes of mice and infection results in splenomegaly and erythroleukemia in susceptible mouse strains (15). Mice that are resistant to FV-induced disease mount early and strong immune responses against the virus, but nevertheless develop lifelong chronic infections. Previously, it was shown that NK cells had anti-retroviral activities during acute infection, but retroviruses evade NK cell recognition (16). For instance, NK cells are able to recognize retrovirus-infected cells via the interaction of the activating receptor natural-killer group 2, member D (NKG2D) and its ligand Retinoic acid early inducible 1 (RAE-1) on target cells, although a virus-mediated inhibition of ligand expression was reported (17, 18). NK cells require several cytokines for their development, survival and activation; however, the cytokines IL-15 and IL-18 mainly influenced the antiviral activity of NK cells against FV-infected target cells and they were predominantly produced by activated macrophages and activated DCs (19). The viral inoculation dose was important for this cytokine-mediated induction of NK cells, because only a high-dose infection induced the antiviral activity of NK cells. Beside IL-15 and IL-18, NK cell activation depends on IL-2 that is predominantly produced by activated CD4^+^ T cells during many infections (5, 7). Interestingly, IL-2 is also the most important activating cytokine of a suppressive CD4^+^ T cell population, called regulatory T cells (Tregs). Tregs are essential for maintaining the peripheral tolerance by preventing autoimmune diseases and limiting chronic inflammation whereas their function can also be associated with progressive effector cell exhaustion and the establishment of chronic infections (20). In FV as well as HIV-1 infection, Tregs were associated with dysfunctional cytotoxic CD8^+^ T cell responses, promoting the development of a chronic infection (21–23). In addition to CD8^+^ T cell suppression, Tregs also suppress NK cell responses by depriving them of IL-2, thereby preventing cytotoxic NK cells from eliminating infected cells (24). As IL-2 is not available for consumption during the very early phase of acute FV infection (up to 3 dpi), we addressed the question whether Tregs were activated during the first days of FV infection and if they contributed to the insufficient activation of NK cells after infection of mice with lower doses of FV. In this work, we demonstrate that NK cells do not show cytotoxicity in the presence of Tregs in mice acutely infected with lower doses of FV. During initial retroviral infection, Tregs became activated and produced immunosuppressive cytokines such as IL-10 and TGF-β. Activated Tregs exert a suppressive activity on NK cells via an IL-10-dependent pathway. Our results highlight a novel aspect of Treg-mediated NK cell regulation during the very early phase of retrovirus infection.

## Material and methods

### Friend virus and infection of mice

Experiments were performed using sex- and age-matched inbred C57BL/6 mice (Harlan Laboratories, Germany) and wild type and transgenic DEREG mice (25). At day 0 of experiments, mice were between 7 and 10 weeks old. For the experiments, the FV complex containing B-tropic Friend murine leukemia helper virus and polycythemia-inducing spleen focus-forming virus was used. The FV stock was prepared as a 15% spleen cell homogenate from susceptible BALB/c mice infected 14 days previously with 3,000 SFFU of FV. Mice were injected intravenously with 0.1 ml phosphate-buffered saline (PBS) containing 20,000 SFFU of FV, if not indicated differently. The virus stock did not contain lactate dehydrogenase-elevating virus. Mice were sacrificed at 3 dpi by cervical dislocation.

### Infectious center assay

Detection of infectious centers was done by 10-fold dilutions of single-cell suspensions onto *Mus dunnis* cells. Co-cultures were incubated for 72 h and fixed with ethanol. *Mus dunni* cells were stained with the F-MuLV envelope-specific monoclonal antibody 720, and developed with a peroxidase-conjugated goat anti-mouse antibody. In a final step, cells were incubated with aminoethylcarbazol for the detection of foci.

### Flow cytometry

Multi-parameter flow cytometry was done with the following antibodies: CD3 (17A2), CD4 (RM4-5), CD11b (M1/70), CD11c (N418), CD49b (DX5), CD69 (H1.2F3), CD80 (16-10A1), CD86 (GL1), F4/80 (BM8), FasL (MFL3), Gr-1 (RB6-8C5), GzmB (GB11), ICOS (7E.17G9), IL-10 (JES6-5H4), KI-67 (SolA15), KLRG-1 (2F1), NK1.1 (PK136), PD-L1 (10F.9G2), Ter119 (Ter119), TGF-β1 (TW7-16B4), TNFα (MP6-T22) and Foxp3 (FjK-16S). For the identification of FV-infected cells a FV protein gp70 (Ab720) Alexa Fluor 647-conjugated antibody was used (26). To exclude dead cells, cells were stained with Zombie UV (Fixable Viability Kit, BioLegend) dye. For gating on lineage-negative (lin^−^) cells, dead cells, T cells and NK cells were excluded from the analysis. Splenocytes were restimulated with ionomycin (500 ng/ml), phorbol myristate acetate (PMA; 25 ng/ml), monensin (1X), and brefeldin A (2 μg/ml) diluted in Iscove's modified Dulbecco's medium (IMDM) buffer at 37°C for 3 h. For intracellular stainings, cells were fixed with Fixation/Permeabilization Solution Kit (BD Biosciences) whereas cells were fixed with Foxp3 Transcription Factor Fixation/Permeabilization kit (Thermofisher) for intranuclear stainings. Data were acquired at LSR II flow cytometer (BD).

### *In vitro* cytotoxicity assay

NK cells were isolated from spleens with the MojoSort Mouse NK cell Isolation Kit (BioLegend) according to the manufacturer's protocol. YAC-1 cells or FBL-3 cells were stained with carboxyfluorescein succinimidyl ester (CFSE, 2.5 μM). Cells were co-incubated in an ET ratio of 25:1. The co-incubation was performed in 96-well U-bottom plates at 37°C in a humidified 5% CO_2_ atmosphere. After 18 h cells were washed and stained with fixable viability dye. Cells were measured immediately at LSR II.

### RNA isolation and real-time PCR

Total RNA was isolated using the DNA/RNA Shield (Zymo research) and the innuPREP RNA mini kit (Analytik Jena). cDNA was synthesized with innoScipt reverse transcriptase (Analytik Jena). Real time-PCR analysis of IL-15 and IL-18 was performed using innuMIX quantitative PCR (qPCR) MasterMix SyGreen (Analytik Jena). Oligonucleotide sequences were ordered at Biomers as follows: for β-actin, 5′-AAATCGTGCGTGACATCAAA-3′ and 5′-CAAGAAGGAAGGCTGGAAAA-3′; IL-15, 5′-CATTTTGGGCTGTGTCAGTG-3′ and 5′-TCTTCAAAGGCTTCATCTGCAA-3′. For the detection of mouse IL-18 Mm-Il18-1-SG QuantiTect primer assay was purchased from Qiagen. The quantitative mRNA levels were determined by using Rotor-Gene Q series software (Qiagen) and were normalized to the β-actin mRNA expression levels.

### NK cell and treg depletion

Mice were injected intraperitoneally with the NK1.1-specific monoclonal antibody PK136 1 day prior FV infection and 1 day after infection to deplete NK cells. More than 90% of NK cells (CD3^−^ CD49b^+^ NK1.1^+^) were depleted in the spleen. To deplete regulatory T cells in transgenic DEREG mice, mice were injected intraperitoneally with DT (0.5 μg, Calbiochem) diluted in PBS at −1 and 1 dpi.

### Neutralization of IL-10 and TGF-β

To neutralize IL-10, mice were injected with 50 μg LEAF Purified anti-mouse IL-10 antibody (JES5-2A5, BioLegend) at day 1, 2, and with 100 μg at day 1. For the neutralization of TGF-β, mice were injected i. p. with 200 μg of InVivoMAb anti-mouse TGF-β (1D11.16.8, BioXCell) every other day starting 1 day prior infection.

### Statistical analyses

Statistical analyses were computed with Graph Pad Prism version 6. Statistical differences between two different groups were determined by the Mann–Whitney test (non-parametric) or unpaired *t*-test (parametric test), depending on the Gaussian distribution. Differences between more than two groups were analyzed by Kruskal–Wallis test (non-parametric) or Ordinary one-way ANOVA (parametric test). If indicated, Rout method was used to remove outliers.

## Results

### NK cells proliferate and become functionally activated in the absence of tregs during early FV infection

**Figure 1:**
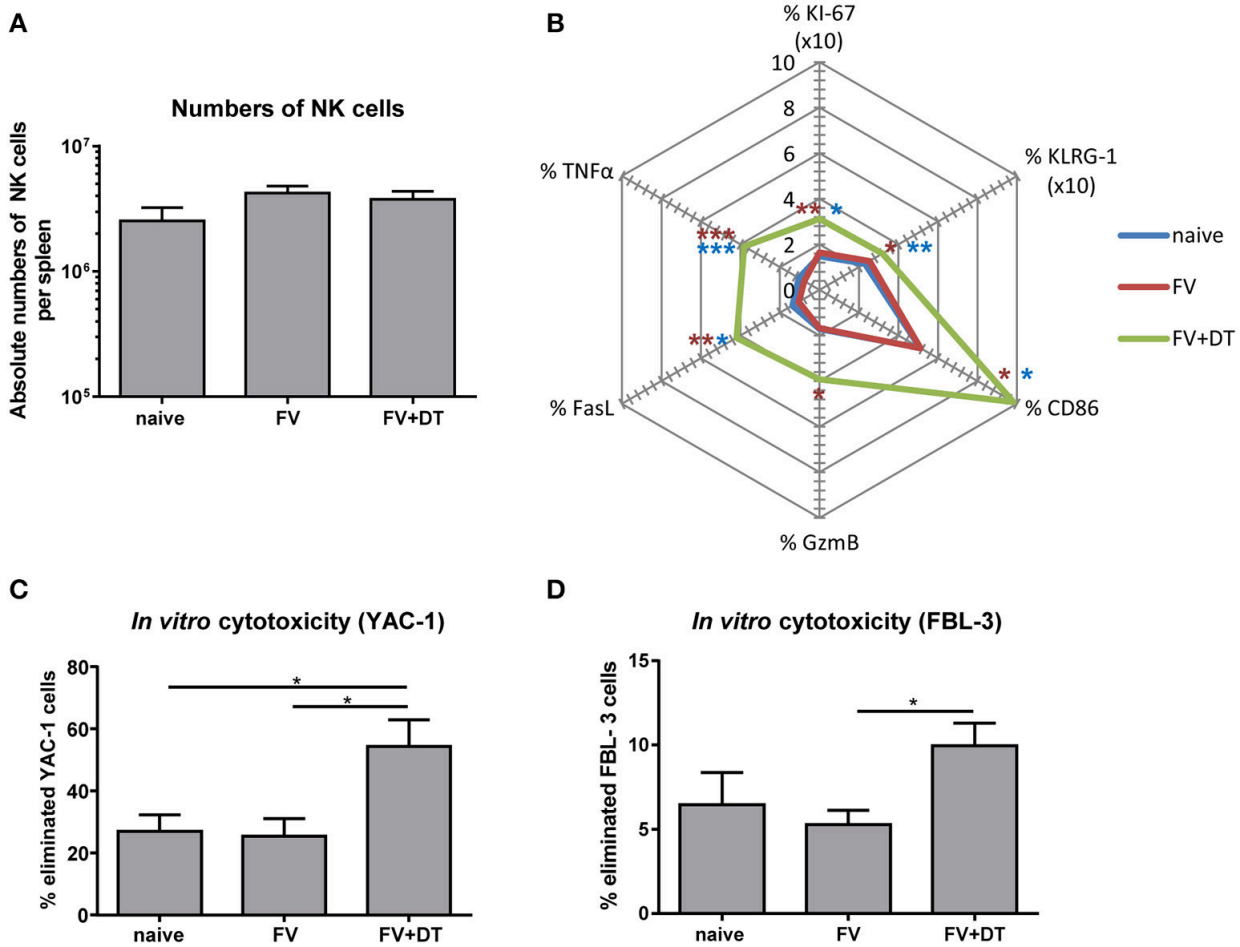
Influence of Tregs on the NK cell phenotype during an acute FV infection. C57BL/6 and DEREG mice were infected with FV and Tregs were depleted by application of diphtheria toxin (DT) into DEREG mice. Splenocytes were harvested at 3 dpi and naïve mice were used as controls. Absolute numbers of splenic NK cells were analyzed by flow cytometry by gating on viable CD3^−^ NK1.1^+^ CD49b^+^ cells **(A)**. Mean values of cell numbers from at least 11 animals are shown, with standard errors of the means (SEM) indicated by error bars (naïve *n* = 11, FV *n* = 16, FV + DT *n* = 16). Effector functions of NK cells were analyzed by KI-67, KLRG-1, CD86, GzmB, FasL, and TNFα and mean percentages are shown in a spider plot **(B)**. At least 8 animals per group from at least three independent experiments were used for the analysis (KI-67, KLRG-1, CD86, GzmB: naïve *n* = 11, FV *n* = 16, FV + DT *n* = 16; FasL, TNFα: naïve *n* = 9, FV *n* = 16, FV + DT *n* = 10). Statistically significant differences were analyzed by Kruskal–Wallis test (KI-67, CD86, GzmB, and FasL) or Ordinary one-way ANOVA (KLRG-1 and TNFα). Differences between groups of naïve mice and Treg-depleted mice (FV + DT) were indicated by blue asterisks whereas differences between the groups of FV-infected animals (FV) compared to groups of Treg-depleted mice (FV + DT) were indicated by red asterisks. The cytotoxicity of NK cells was analyzed by co-incubation of isolated NK cells with CFSE-labeled YAC-1 cells (**C**; naïve *n* = 11, FV *n* = 11, FV + DT *n* = 6) or FV-induced FBL-3 cells (**D**; naïve *n* = 7, FV *n* = 11, FV + DT *n* = 7). After co-incubation, cells were stained for viability and immediately analyzed by flow cytometry. At least 6 animals per group from at least three independent experiments were used for the analysis. Statistically significant differences were analyzed by Kruskal–Wallis test are indicated as follows: *p < 0.05, **p < 0.01, ***p < 0.001.

Since application of diphtheria toxin (DT) into transgenic DEREG mice results in a depletion of up to 90% of Tregs (25), we used these mice to analyze the regulation of NK cells by Tregs. To address the question whether Tregs suppress NK cell functions during initial FV infection (3 dpi), we depleted Tregs and subsequently determined NK cell numbers and their phenotype. All infection experiments were performed with a medium dose of FV inoculum, which we previously demonstrated to induce only very weak NK cell responses during acute infection (19). We did not detect any changes in the absolute numbers of CD3^−^ CD49b^+^ NK1.1^+^ cells in the spleen after FV infection (3 dpi) nor increased NK cell numbers after Treg depletion (Figure 1A). However, Treg depletion increased the percentage of proliferating (KI-67), activated and matured (KLRG-1), effector phenotype (CD86, GzmB, and FasL) and cytokine producing (TNFα) NK cells during acute infection (Figure 1B). To test the cytotoxic potential of NK cells against target cells *in vitro*, we co-incubated isolated NK cells with YAC-1 cells and determined their killing capacity (Figure 1C). We did not detect any increased killing of target cells after FV infection in comparison to naïve mice whereas the ablation of Tregs resulted in a significant increase of NK cell-mediated target cell elimination. Similarly, we analyzed the killing of the FV-induced tumor cell line FBL-3 (Figure 1D), which expresses FV antigens but does not produce infectious virus. Again, we detected a significantly augmented elimination of FBL-3 target cells when using NK cells from infected, Treg-ablated mice compared to the only FV group. Thus, the absence of suppressive Tregs improved NK cell proliferation, activation and cytotoxicity during acute FV infection.

### Activation of DCs and macrophages correlates with higher levels of IL-15 after depletion of tregs during acute FV infection

**Figure 2:**
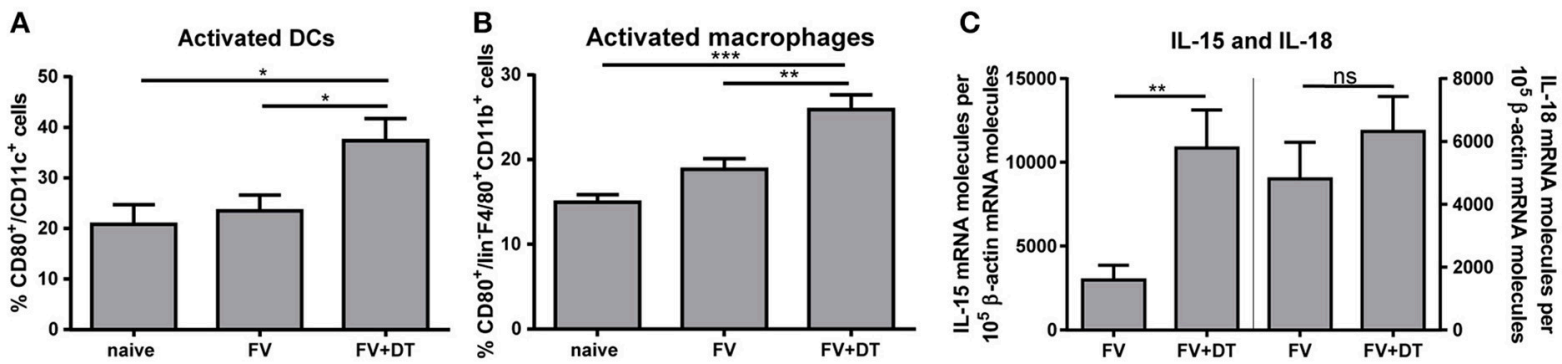
IL-15 and IL-18 mRNA expression levels and activation of DCs and macrophages in FV-infected mice. DEREG mice were infected with FV and depleted for Tregs by repeated injections of DT. Mice were sacrificed at 3 dpi and uninfected mice were used as controls. Spleen cells were analyzed for the activation of CD11c^+^ DCs (**A**; naïve *n* = 10, FV *n* = 15, FV + DT *n* = 12) and F4/80^+^ CD11b^+^ macrophages (**B**; naïve *n* = 10, FV *n* = 15, FV + DT *n* = 12) by detection of the surface molecule CD80. At least 10 animals per group from at least 3 independent experiments were used for the Ordinary one-way ANOVA. mRNA expression levels of IL-15 (left; FV *n* = 16, FV + DT *n* = 14) and IL-18 (right; FV *n* = 16, FV + DT *n* = 14) were analyzed in splenocytes of FV-infected (FV) and Treg-depleted mice (FV + DT) by quantitative real-time PCR **(C)**. The housekeeping gene β-actin was amplified for each sample as an internal standard and for normalization. Samples were collected from at least three independent experiments and were run in duplicate. Outliers were removed using the Rout method. Statistically significant differences were analyzed by Mann-Whitney test and are indicated by *p < 0.05, **p < 0.01, ***p < 0.001. Mean values are displayed, with SEM indicated by error bars.

Activation of NK cells depends on diverse signals such as cytokines and cell-to-cell contacts with myeloid cells (2, 11, 12). Several bidirectional interactions between NK cells and DCs or between NK cells and macrophages were reported (10, 19, 27). Beside NK cells, also Tregs are able to interact with DCs and macrophages. Tregs can block the maturation and functions of DCs and macrophages, e. g. through the ligation of CTLA-4 with CD80 and/or CD86, via binding of LAG3 to MHC II molecules or by the Fas/FasL pathway (28, 29). To address the question whether Tregs suppress the activation of macrophages and DCs, hence indirectly regulate NK cell functions, we analyzed the APC activation by detection of the CD80 co-stimulatory molecule after acute FV infection. FV infection alone did not result in increased percentages of activated APCs whereas the depletion of Tregs during FV infection resulted in an increased percentage of CD80^+^ DCs (Figure 2A) and macrophages (Figure 2B). In a previous study, we demonstrated that during the acute phase of FV infection activated DCs and macrophages were sources of the NK cell-stimulatory cytokines IL-15 und IL-18 (19). Because macrophages and DCs are able to produce large amounts of IL-15 and IL-18, we analyzed IL-15 and IL-18 mRNA levels in splenocytes from FV-infected and Treg depleted animals during initial infection. Interestingly, we did not detect a significant increase in IL-18 mRNA levels but we found a substantially augmented mRNA level for IL-15 in Treg-ablated mice (Figure 2C). Taken together, Tregs suppress the activation of DCs and macrophages that correlates with reduced mRNA levels for IL-15 during the first days of FV infection.

### Treg deletion results in more efficient spread of FV, but activated NK cells control virus replication

**Figure 3:**
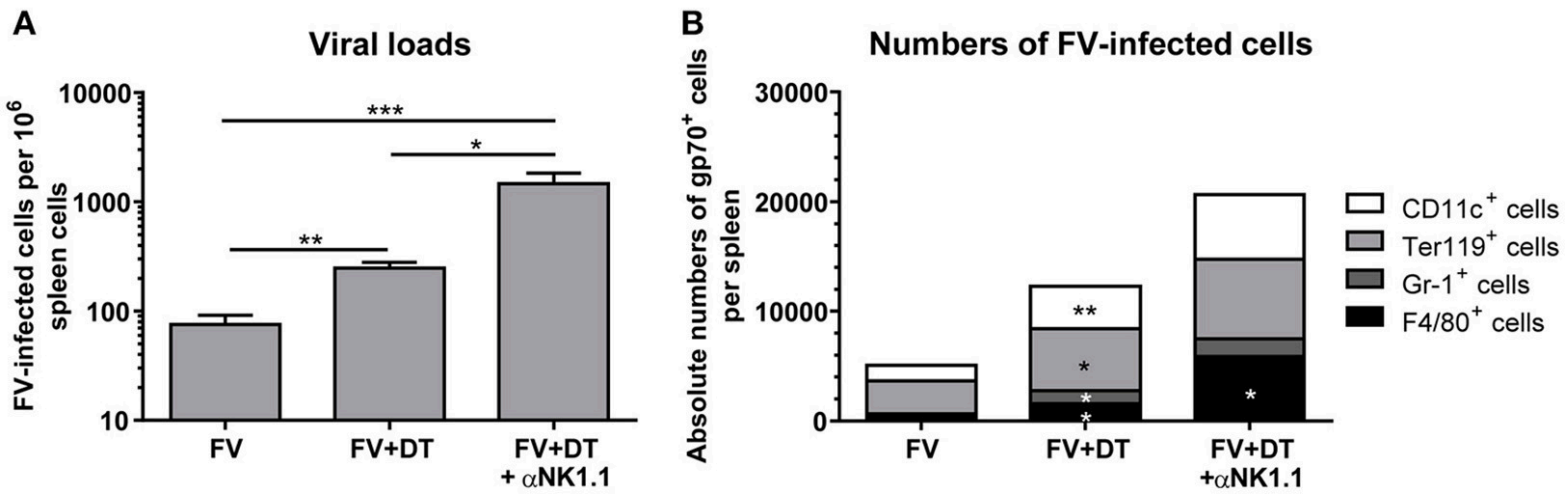
Viral loads and numbers of FV-infected cells after Treg depletion. Mice were infected with FV and spleens were harvested at 3 dpi. In one group, Tregs were depleted by repeated injections of DT into DEREG mice (FV + DT). Additionally, one group of Treg-depleted mice were ablated for NK cell by repeated injections with the NK1.1-specific monoclonal antibody PK136 (FV + DT + αNK1.1). Viral loads were analyzed by an infectious center assay and results are shown in **(A)** by mean values, with SEM indicated by error bars. At least 6 animals per group from at least two independent experiments were used for the analysis (FV *n* = 19, FV + DT *n* = 20, FV + DT + αNK1.1 *n* = 6). Outliers were removed using the Rout method. Statistically significant differences were analyzed by the Kruskal–Wallis test. **(B)** Absolute numbers of FV-infected cells were analyzed by the expression of FV gp70 antigen on the cell surface of CD11c^+^ DCs, Ter119^+^ erythroblasts, Gr-1^+^ CD11b^+^ granulocytes, and F4/80^+^ CD11b^+^ macrophages (FV *n* = 9, FV + DT *n* = 9, FV + DT + αNK1.1 *n* = 6). Statistically significant differences between the group of FV-infected and FV + DT group or comparison between FV + DT and FV + DT + αNK1.1 were analyzed by Mann-Whitney test and are indicated as follows: *p < 0.05, **p < 0.01, ***p < 0.001.

To analyze the influence of Treg depletion on the viral replication and spread, we measured viral loads in spleens of FV-infected mice (3 dpi) with an infectious center assay (Figure 3A). Depletion of Tregs (FV + DT) resulted in a more than 3-fold increase of viral loads in comparison to the only FV-infected group (FV). NK cell depletion in animals infected with 20,000 SFFU of FV, had no influence on viral loads (19). The question was, why viral loads increased at 3 dpi after Treg depletion and one possible explanation was that Tregs also control the activation of the target cells for FV. As target cells for FV infection, erythroid precursor cells, monocytes, granulocytes, T cells and B cells were described, and FV is known to efficiently infect activated and dividing cells (30, 31). We therefore analyzed cell subsets, which are potential targets for early FV infection. To this end, we used a viral envelope protein gp70-specific antibody (Ab720) that allows the detection of FV-infected cells (26). Absolute numbers of infected erythroid precursor cells (Ter119^+^) and granulocytes (Gr-1^+^) as well as macrophages (F4/80^+^) and DCs (CD11c^+^) were analyzed by multi-parameter flow cytometry and calculated for whole spleens. Initial FV infection resulted in moderate numbers of gp70^+^ cells whereas depletion of Tregs led to a significant increase in gp70^+^ DCs, erythroblasts, granulocytes and macrophages (Figure 3B). Thus, reduced suppression of target cell activation due to Treg depletion resulted in enhanced infection levels during acute FV infection.

Because we detected increased viral loads after Treg depletion but, counter-intuitive, also an increased cytotoxic activity of NK cells, we determined whether or not NK cell activation had an effect on the levels of virus replication. Additional depletion of NK cells led to a mean viral burden of 1,474 FV-infected cells per million cells in comparison to 248 infected cells in the FV + DT group (Figure 3A). In addition, NK cell depletion in Treg-ablated mice substantially increased numbers of gp70^+^ macrophages (Figure 3B). Thus, activated NK cells reveal an anti-retroviral function and efficiently reduce numbers of infected cells in the absence of Tregs.

### Tregs become activated during acute FV infection and produce IL-10 and TGF-β

**Figure 4:**
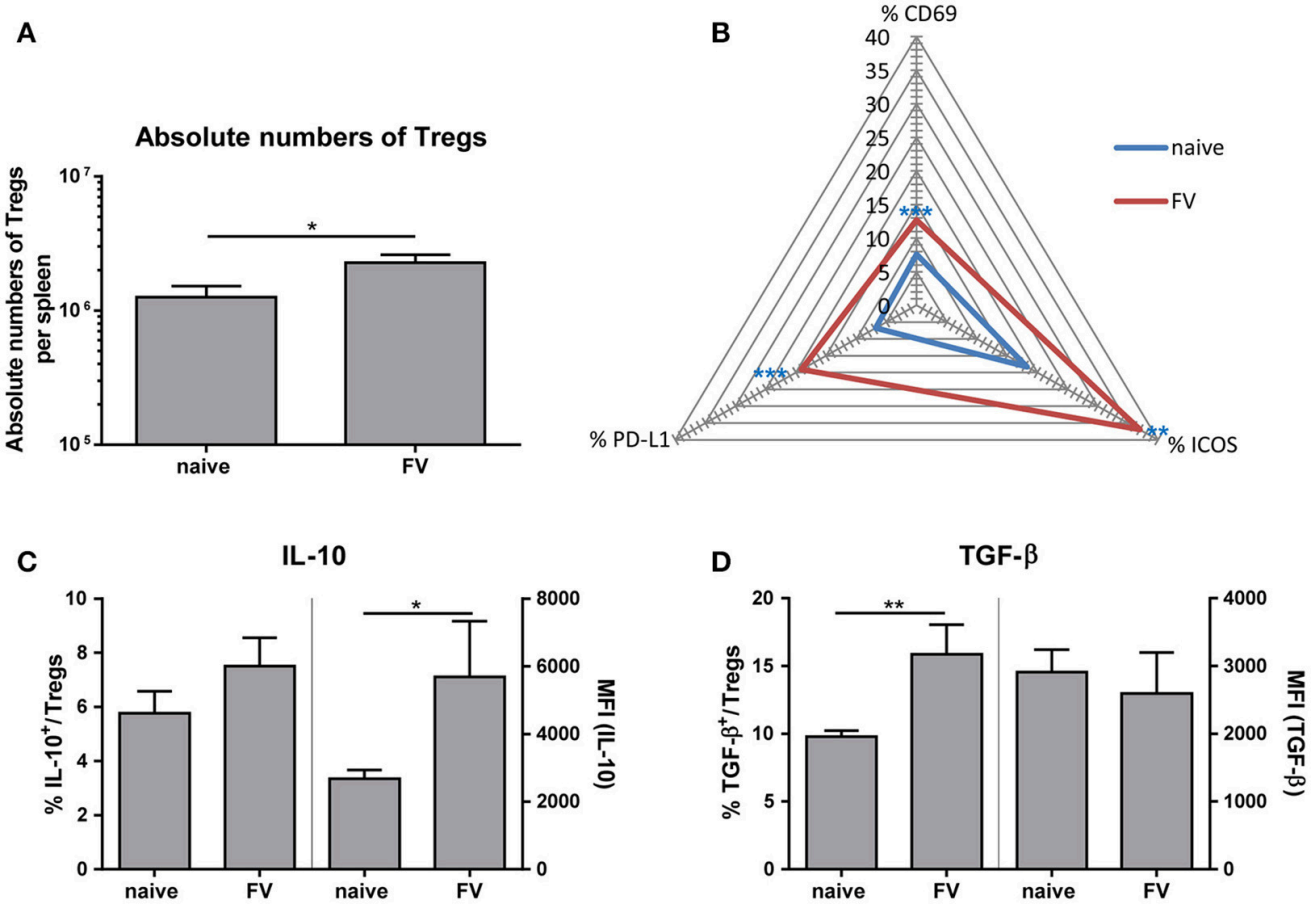
Phenotype of Tregs during an acute FV infection. Mice were infected with FV and spleen cells were analyzed at 3 dpi. Uninfected mice were used as control. Absolute numbers of CD4^+^ Foxp3^+^ Tregs were analyzed in **(A)** (naïve *n* = 18, FV *n* = 26) and are displayed by mean values (±SEM). Statistically significant differences were analyzed by Mann-Whitney test. Activation of Tregs was analyzed by multi-parametric flow cytometry and mean percentages are shown in a spider plot **(B)**. At least 6 values from two independent experiments were used for the analysis (CD69: naïve *n* = 14, FV *n* = 19; PD-L1 and ICOS: naïve *n* = 8, FV *n* = 6). Statistically significant differences were analyzed by Mann-Whitney test (CD69) or unpaired *t*-test (ICOS, PDL-1). The percentages (left axis) as well as the mean fluorescence intensity (MFI, right axis) of IL-10^+^ or TGF-β^+^ Tregs are shown in **(C)** (naïve *n* = 9, FV *n* = 6) and **(D)** (naïve *n* = 9, FV *n* = 6). A minimum of 6 animals from two independent experiments were used for the analysis. Mean values are displayed, with SEM indicated by error bars. Statistically significant differences were analyzed by an unpaired *t*-test and are indicated as follows: *p < 0.05, **p < 0.01, ***p < 0.001.

On one hand, Tregs are essential for maintaining self-tolerance and homeostasis but on the other hand they can contribute to viral spread and chronic infections. However, whether these suppressive cells control the initial immune response to acute viral pathogens needs to be further elucidated. To study the phenotype of Tregs during acute infection, we first determined the absolute numbers of CD4^+^ Foxp3^+^ Tregs in the spleen at 3 dp FV infection (Figure 4A). We detected almost two-times higher numbers of Tregs in FV-infected spleens compared to naïve organs. Furthermore, we analyzed the activation of Tregs by the early activation marker CD69, the inducible co-stimulator ICOS (CD278) and the Programmed cell death ligand 1 (PD-L1, CD274) that are upregulated on activated T cells (32, 33). Compared to the naïve group, FV infection substantially increased the percentage of CD69^+^, ICOS^+^ and PD-L1^+^ Tregs at 3 dpi, indicating early Treg activation and expansion (Figure 4B). Treg are able to suppress immune cells through several mechanisms such as metabolic disruption, modulation of DCs or release of inhibitory cytokine (29). Particularly the inhibitory cytokines IL-10 and TGF-β have been the focus of considerable attention as mediators of Treg suppression. Therefore, we analyzed these molecules in CD4^+^ Foxp3^+^ Tregs during acute FV infection via multi-parameter flow cytometry. Although we did not detect significant changes in the percentages of IL-10 expressing Tregs in FV infection compared to naïve mice, we showed a significant higher Mean fluorescence intensity (MFI) of IL-10 in Tregs from the group of FV-infected animals (Figure 4C). TGF-β is first synthesized as precursor TGF-β protein and then non-covalently linked to the latency-associate peptide (LAP) (34). Involving also other proteins, this protein complex increases the secretion efficiency of TGF-β and targets the complex to the extracellular matrix (35, 36). Here, we used an anti-mouse LAP antibody to detect TGF-β1^+^ Tregs in the spleen of naïve and FV-infected mice (Figure 4D). The analysis of TGF-β revealed an increased percentage of TGF-β1^+^ Tregs but no changes in the MFI for TGF-β in Tregs were detected. Thus, we demonstrated that IL-10^+^ Tregs produced more IL-10 and additional Tregs initiated the production of TGF-β after a few days of FV infection.

### Neutralization of IL-10 and TGF-β during acute FV infection has a direct effect on the NK cell phenotype, which is not mediated through activated DCs or macrophages

**Figure 5:**
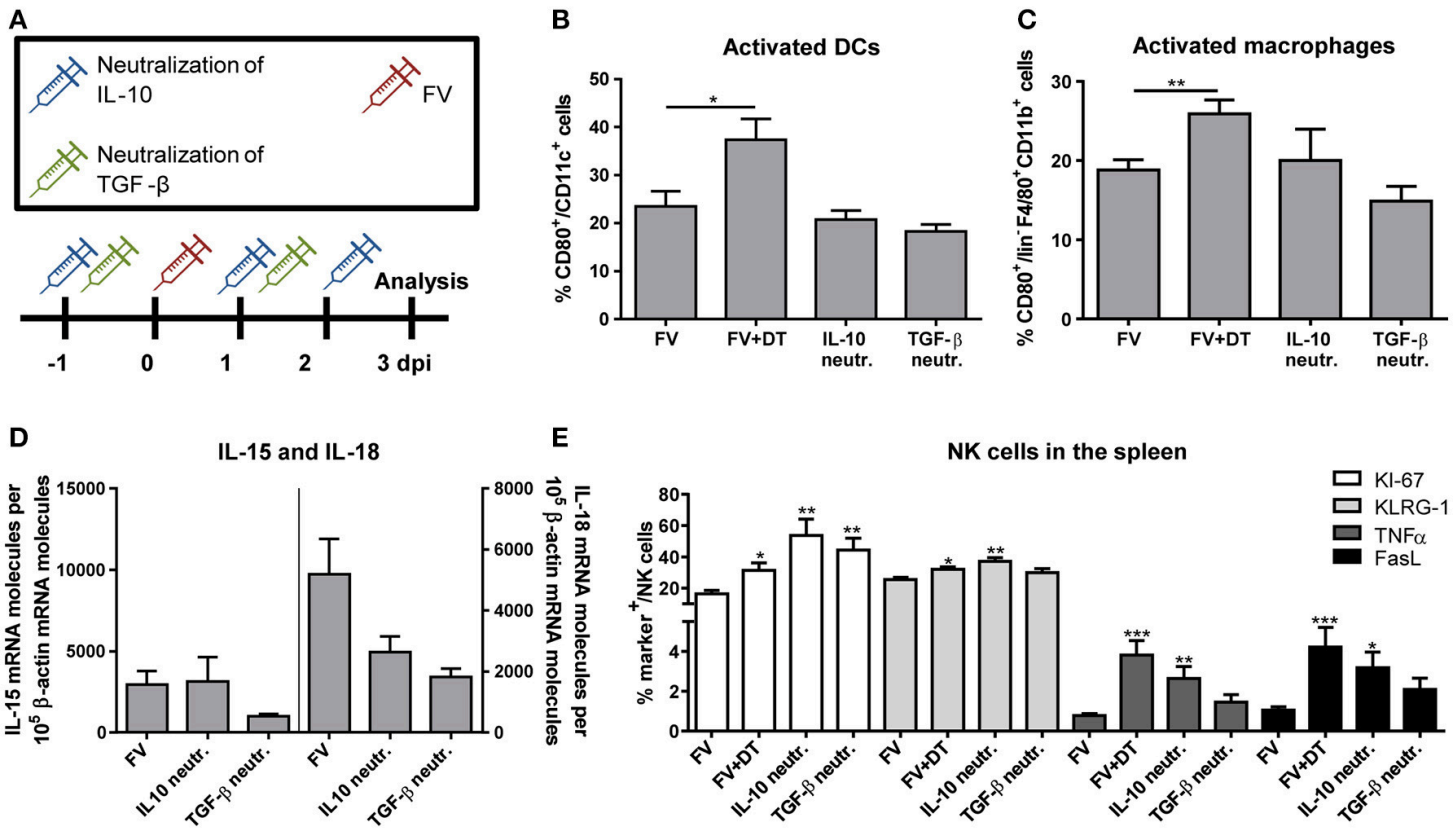
Influence of IL-10 and TGF-β neutralization on innate immune cells during an acute FV infection. Mice were infected with FV and groups of infected mice were also neutralized for IL-10 (IL-10 neutr.) or TGF-β (TGF-β neutr.) as displayed in **(A)**. Spleens of FV-infected mice were removed at 3 dpi and single cell suspensions were prepared. Activation of DCs **(B)** and macrophages **(C)** were analyzed by flow cytometry (**B,C**: IL-10 neutr. *n* = 7; TGF-β neutr. *n* = 6). mRNA expression levels of IL-15 (left; IL-10 neutr. *n* = 13; TGF-β neutr. *n* = 12) and IL-18 (right; IL-10 neutr. *n* = 14; TGF-β neutr. *n* = 12) were analyzed in spleen cells of FV-infected (FV) and IL-10- or TGF-β-neutralized mice by quantitative real-time PCR **(D)**. The housekeeping gene β-actin was amplified for each sample as an internal standard and for normalization. Samples were collected from two independent experiments and were run in duplicate. One outlier was removed using the Rout method. In E, the effector phenotype of NK cells was determined by the expression of KI-67 (white bars, *n* = 7), KLRG-1 (light gray bars), TNFα (gray bars) and FasL (black bars). At least 6 animals were used from at least two independent experiments (IL-10 neutr. *n* = 7; TGF-β neutr. *n* = 6). Statistically significant differences were analyzed by ordinary one-way ANOVA **(B,C)** or Kruskal–Wallis **(E)** test and are indicated as follows: *p < 0.05, **p < 0.01, ***p < 0.001. Percentages are shown as mean ± SEM.

Our observation that Tregs produced immunosuppressive cytokines and NK cells were inhibited by Tregs raised the question, whether this inhibition might be mediated via the cytokines IL-10 and/or TGF-β. Therefore, we neutralized IL-10 or TGF-β by repeated injections of neutralizing antibodies as it is shown in Figure 5A. In a previous study, we have demonstrated that activated macrophages and DCs efficiently augment the antiviral activity of NK cells (19). Thus, we first addressed the question whether IL-10 and TGF-β suppressed the activation of these APCs. Figures 5B,C show that we did not detect an IL-10- or TGF-β-mediated suppression of DC or macrophage activation, measured by CD80 expression. In addition, IL-10 or TGF-β neutralization did not significantly affect levels of mRNA for IL-15 or IL-18 in splenocytes (Figure 5D). In a next step, we addressed the question whether the Treg-produced cytokines suppressed the NK cell activity. Indeed, we detected a significantly increased percentage of proliferating NK cells in FV-infected mice after IL-10- and TGF-β-neutralization. Beside the proliferation (KI-67), we analyzed activated (KLRG-1) NK cells, determined the percentage of cytokine-producing (TNFα) NK cells and measured the cytotoxicity-associated marker FasL (Figure 5E). Interestingly, only IL-10 neutralization resulted in increased percentages of KLRG-1^+^, FasL^+^ and TNFα^+^ NK cells, but not TGF-β neutralization. Collectively, the data indicate that IL-10 directly suppressed NK cell proliferation and functions during initial FV infection, but did not influence the activation of macrophages and DCs.

## Discussion

Despite significant success in therapeutic strategies to combat HIV, 36.7 million people worldwide were still living with HIV in 2016 (37). Although treatment successfully controls the viral replication, HIV remains persistent and cannot be eradicated. Beside the development of HIV vaccines, basic research is necessary to get deeper insights into the host immune defense and to find a cure or a functional cure for retroviral infections. In this study, we demonstrate a suppressive effect of Tregs on the antiviral NK cell response during the first days of acute retrovirus infection through a mechanism involving the immunosuppressive cytokine IL-10, but not TGF-β.

During immune homeostasis, Tregs prevent autoimmune diseases and inflammation and constitute approximately 5–10% of CD4^+^ T cells in rodents. In the case of infections, Tregs are necessary to control cytotoxic T cell responses to avoid immunopathology, but they also enable viral spread and the establishment of chronic infections (23, 38). Tregs are known to suppress cytotoxic CD8^+^ T cells in several chronic infections with viruses such as HIV, hepatitis B, hepatitis C or FV (21, 22, 39, 40). Importantly, numbers of Tregs are a key parameter for the suppression of immune cells. We know from previous studies in uninfected mice (41) or at the peak of Treg activation in the FV model (12 dpi) (24) that Tregs inhibit NK cell functions by depriving them of IL-2. During later periods of infections, CD4^+^ T cells produce increased amounts of IL-2, which is predominantly consumed by Tregs due to their expression of the high-affinity IL-2 receptor complex (5, 7). However, no activation of CD4^+^ T cells was detected during the early phase of FV infection (7) and no study investigated the influence of Tregs on very early immunological events during an retroviral infection. In contrast to the other studies, the current study provides intriguing insights into very early retroviral infection (3 dpi) and the interaction of the virus with immune cells, which is almost impossible to analyze in clinical studies.

Tregs are known to suppress target cells not only through metabolic disruption such as IL-2 deprivation, but also via induction of apoptosis by the production of cytotoxic molecules (e. g. GzmA, GzmB), inhibition of DC functions and maturation (e. g. CTLA-4—CD80/CD86; LAG3—MHCII) and secretion of inhibitory cytokines (29). Tregs are associated with the release of anti-inflammatory cytokines such as IL-10 and TGF-β that also promote the Treg generation and prevent disproportionate inflammation and autoimmune manifestations (42). In this study, we showed increased proportion of TGF-β^+^ Tregs during the initial phase of retroviral infection. We demonstrated that TGF-β is not the key mediator of Tregs during the initial retroviral infection. Indeed, the neutralization of TGF-β resulted in an increased proliferation of NK cells but did not improve effector functions of NK cells. Contradictory, several research groups revealed a contact-dependent Treg-mediated suppression of NK cells by TGF-β but these studies were performed without any viral background (43–45).

It has been shown that IL-10 produced by Tregs regulates the expansion of T cells, thereby preventing auto-aggressive cell expansions (46). In the current study we show that Tregs increased the expression of IL-10 during acute FV infection that is supported by significantly decreased IL-10 mRNA levels in splenocytes of Treg-depleted mice (data not shown). Interestingly, IL-10 neutralization augmented the NK cell proliferation, activation, cytotoxicity and cytokine production, but did not alter the activation of APCs. In addition, IL-10 did not influence the production of the NK cell-stimulating cytokines IL-15 or IL-18. It is therefore very likely that the immunosuppressive cytokine IL-10 directly suppressed the NK cell activity in FV-infected mice. These results are in line with previous studies that described IL-10 as inhibitor of NK cells activation and cytokine production (47, 48). In these studies, this inhibition involved the blockade of the NK cell-stimulatory IL-12 and IL-18 production by accessory cells (47, 48). However, IL-12 does not play a major role in the acute FV infection (19). In this study, we showed that the IL-10-mediated inhibition has a direct influence on NK cells and has no indirect effect on the activation of DCs or macrophages. Nevertheless, the mechanism of APC suppression by Tregs during early FV infection is still unsolved but might be possible through cell-to-cell contacts.

In the acute HIV and FV infection, NK cells contribute to the viral control by eliminating infected target cells and by the production of cytokines (16, 49). By using different doses of FV for challenge infection, we previously demonstrated the importance of initial viral titers for the initiation of efficient NK cell responses during acute infection (3 dpi) (19). Only high-dose FV infection resulted in increased activation and IL-15/IL-18 production by macrophages and DCs that stimulated antiviral functions of NK cells. Our observations that lower doses of FV did not induce an efficient NK cell response raised the question whether Tregs are involved in the NK cell unresponsiveness (3 dpi). The lower dose infection of mice is of outstanding physiological relevance as it reflects the typical transmission of HIV particles in humans. In the current study we show that Tregs were increased in numbers and activation during initial lower dose FV infection. This increased activity resulted not only in the suppression of NK cells but also in the inhibition of APC activation. Here, we were interested whether the phenotype of Tregs was also sensitive to different viral doses. Interestingly, our current data revealed no changes in Treg numbers as well as activation after infection with lower and high viral doses (Supplementary Figure 1). This might reflect the strong Treg suppression of NK cells during the infection with lower viral doses as infection did not result in increased percentages of activated innate immune cells. In contrast, infections with high viral titers strongly increased numbers, improved the activation of APCs and augmented the release of NK cell-stimulatory cytokines that resulted in increased cytotoxic functions of NK cells (19).

APCs are essential producers of NK cell-stimulatory cytokines such as IFNα, IL-12, IL-15 and IL-18 (2, 3). We previously demonstrated low IFNα and IL-12 concentrations but highlighted the strong influence of IL-15 and IL-18 in acute FV infection (19); hence, we also determined levels of IL-15 and IL-18 in Treg-ablated mice in the spleen. After Treg depletion an increased concentration of IL-15 mRNA molecules were detected whereas we did not detect augmented levels of IL-18, pointing out a substantial role of IL-15 for the NK cell activation during early FV infection. IL-15 is mainly produced by myeloid cells and has several immunoregulatory functions. Beside the essential role of IL-15 during the NK cell development and differentiation, it is an important survival factor for peripheral NK cells (50). IL-15 has similar biologic properties as IL-2, although it minimally promotes the activation and maintenance of Tregs (51).

Overall, the data from our studies show different conditions of NK cell activation during FV infection. The current study shows that NK cells were directly suppressed by Tregs in an IL-10-dependent manner during early FV infection (3 dpi). NK cells were activated at high inoculation dose and no suppressive effect of Tregs was detected (3 dpi) (19). High viral titers resulted in an increased activity of macrophages and DCs that produced huge amounts of the NK cell-stimulatory cytokines IL-15 and IL-18 (19). During the later phase of FV infection (12 dpi), the NK cell cytotoxicity was suppressed by Tregs through IL-2 deprivation (24). In the transition phase to chronic infection, NK cells themselves exhibited regulatory functions by decreasing the function of cytotoxic CD8^+^ T cells (52).

In this study, we provide fascinating insights into the early activity of suppressive Tregs that resulted in impaired cytotoxicity of NK cells toward retroviral infections. Our data clearly show the Treg-mediated brake of the innate immune system, which might be an interesting target in future therapies.

## Ethics statement

Animal experiments were executed in strict accordance with the German regulations of the Society for Laboratory Animal Science (GV-SOLAS) and the European Health Law of the Federation of Laboratory Animal Science Associations (FELASA). North Rhine-Westphalia State Agency for Nature, Environment and Consumer Protection (LANUV) approved all experiments and protocols.

## Author contributions

EL-S designed and performed the experiments, analyzed the data, participated in the statistical analysis, and wrote the paper. AM and SS performed several experiments. UD designed the experiments and wrote the paper.

### Conflict of interest statement

The authors declare that the research was conducted in the absence of any commercial or financial relationships that could be construed as a potential conflict of interest.
