# Supplementary material for: The Cytotoxic Activity of Natural Killer Cells Is Suppressed by IL-10+ Regulatory T Cells During Acute Retroviral Infection
Source: Front Immunol. 2018 Aug 27;9:1947. doi: 10.3389/fimmu.2018.01947 (PMC6119693; doi:10.3389/fimmu.2018.01947)
Supplement: Supplementary file 1 [file Data_Sheet_1.docx]

Supplementary Material

The cytotoxic activity of natural killer cells is suppressed by IL-10^+^ regulatory T cells during acute retroviral infection

Elisabeth Littwitz-Salomon*, Anna Malyshkina, Simone Schimmer, Ulf Dittmer

*** Correspondence:** Corresponding Author: [Elisabeth.Littwitz@uni-due.de](mailto:Elisabeth.Littwitz@uni-due.de)

**
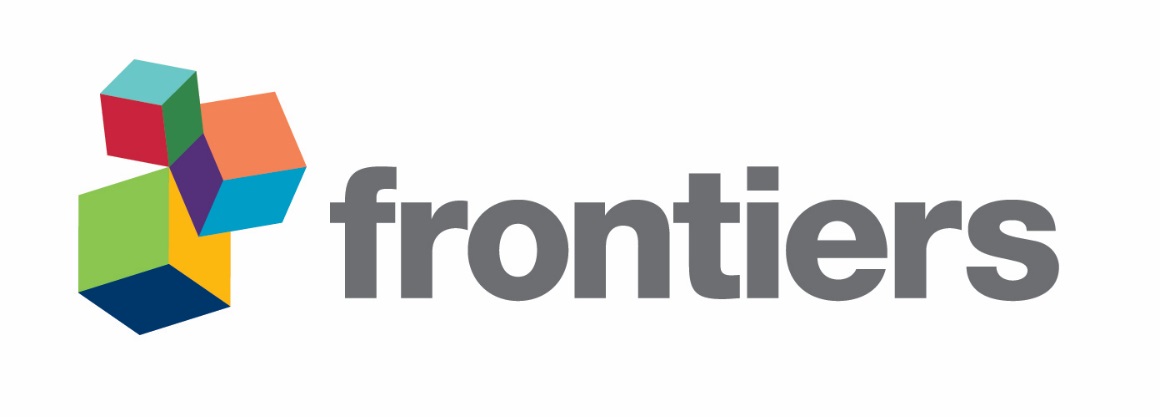
**

**
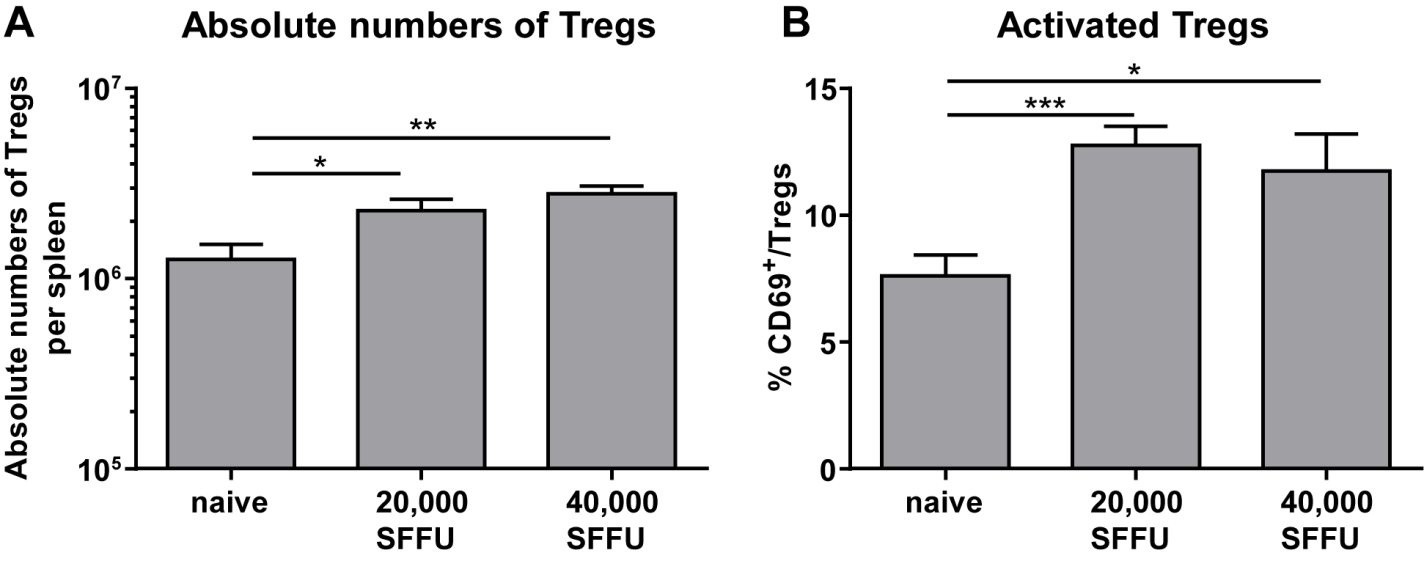
**

**Supplementary Figure 1: Numbers and activation of Tregs during high- and medium-dose of FV-infection**

Mice were infected with medium (20,000 SFFU) or high dose (40,000 SFFU) of FV. Uninfected mice were used as control. At 3 dpi, single cell suspensions of spleen cells were prepared and counted. Absolute numbers of Tregs (A) and activation of Tregs (B) were analyzed by multi-parametric flow cytometry and are shown as mean values, with SEM indicated by error bars. At least 8 mice per group from at least 4 independent experiments were used for the analysis (A: naïve n=18; 20,000 SFFU n=26; 40,000 SFFU n=15; B: naïve n=14; 20,000 SFFU n=19; 40,000 SFFU n=8). Statistically significant differences were determined by Kuskal-Wallis test (A) or ordinary one-way ANOVA (B) and are indicated by * p < 0.05, ** p < 0.01 and *** p < 0.001.
